# HOW DOES SOCIAL INTEGRATION SHAPE HEALTHCARE USE AMONG OLDER ADULTS IMMIGRANTS?

**DOI:** 10.1093/geroni/igae098.4209

**Published:** 2024-12-31

**Authors:** Aditi Patel, Sungjae Hong, Shannon Mejia

**Affiliations:** University of Illinois at Urbana Champaign, Urbana, Illinois, United States; University of Illinois at Urbana-Champaign, Champaign, Illinois, United States; University of Illinois Urbana-Champaign, Urbana, Illinois, United States

## Abstract

Immigrant populations often face significant health challenges due to language or financial barriers, creating unique vulnerabilities that impact community health. This study focuses on social integration among immigrant older adults, which indicates how a relationship between an individual and their community is built and maintained. The aim is to understand how social integration, which has shown varying support for healthcare access, affects immigrant older adults and their use of novel healthcare measures like telehealth. We interviewed 22 Korean immigrant older adults living in the Chicago Metropolitan Area. The interviews covered respondents’ life experiences, focusing on affiliated communities and healthcare experiences. We conducted a content analysis to explore approaches to developing social integration and its implications for the healthcare experience. Our analysis compared the healthcare and telehealth experiences by the types of communities they belonged to and to what extent they felt affiliated with it. The analysis revealed three findings. First, strong affiliations in Korean communities, such as the Korean church, led to access to healthcare information, like available healthcare resources. Second, telehealth services were not associated with the social integration of immigrant older adults because they adopted telehealth during the COVID-19 pandemic based on healthcare providers’ recommendations. Third, strong affiliations with adult children supported the continued utilization of telehealth services in the years that followed the COVID-19 pandemic. These findings highlight the critical role of social networks and social integration in shaping healthcare decisions, offering valuable insights for developing strategies to improve telehealth accessibility and support services for immigrant populations.

